# A Novel Injectable Magnesium/Calcium Sulfate Hemihydrate Composite Cement for Bone Regeneration

**DOI:** 10.1155/2015/297437

**Published:** 2015-06-03

**Authors:** Shanchuan Zhang, Ke Yang, Fuzhai Cui, Yi Jiang, Lingling E, Baohua Xu, Hongchen Liu

**Affiliations:** ^1^Center of Stomatology, China-Japan Friendship Hospital, Beijing 100029, China; ^2^Institute of Stomatology, General Hospital of Chinese PLA, Beijing 100853, China; ^3^Specialized Materials and Devices Division, Institute of Metal Research, Chinese Academy of Sciences, Shenyang 110016, China; ^4^Advanced Materials Laboratory, Tsinghua University, Beijing 100084, China; ^5^Department of Geriatric Dentistry, General Hospital of Chinese PLA, Beijing 100853, China

## Abstract

*Objective.* A novel injectable magnesium/calcium sulfate hemihydrate (Mg/CSH) composite with improved properties was reported here. *Methods.* Composition, setting time, injectability, compressive strength, and bioactivity in simulated body fluid (SBF) of the Mg/CSH composite were evaluated. Furthermore, the cellular responses of canine bone marrow stromal cells (cBMSCs) and bone formation capacity after the implantation of Mg/CSH in tibia defects of canine were investigated. *Results.* Mg/CSH possessed a prolonged setting time and markedly improved injectability and mechanical property (*p* < 0.05). Mg/CSH samples showed better degradability than CSH in SBF after 21 days of soaking (*p* < 0.05). Moreover, the degrees of cell attachment, proliferation, and capability of osteogenic differentiation on the Mg/CSH specimens were higher than those on CSH, without significant cytotoxicity and with the increased proliferation index, ALP activity, and expression levels of integrin *β*1 and Coll I in cBMSCs (*p* < 0.05). Mg/CSH enhanced the efficiency of new bone formation at the tibia defect area, including the significantly elevated bone mineral density, bone area fraction, and Coll I expression level (*p* < 0.05). *Conclusions.* The results implied that this new injectable bone scaffold exhibited promising prospects for bone repair and had a great potential in bone tissue engineering.

## 1. Introduction

Bone defects are usually caused by trauma and nonunion, and autologous bone graft is commonly known as a gold standard in reconstruction of bone defects [1]. In the USA alone, approximately 1.6 million bone graft operations are performed for the treatment of bone defects every year [2]. However, the clinical use of autologous bone graft is accompanied by size limitations and a considerable donor site morbidity, like bleeding, hematoma, infection, and chronic pain. Currently, a variety of biomaterials, like acrylate-, calcium phosphate-, or apatite-based bone cements, and porous composites, are being used for reconstruction of bone defects [3].

Reportedly, calcium sulfate hemihydrate (CaSO_4_·1/2H_2_O; CSH) has long been used in clinic as a bone regeneration material, because it is characterized by low curing temperature (about 30°C), rapid setting, excellent biocompatibility without inducing an inflammatory response, and promotion of bone healing [4–8]. However, the currently prepared CSH cement still has some limitations, which significantly limit its clinical applications. The solidified CSH paste has poor mechanical properties, which fails to provide constant long-term mechanical support for the defect site [9, 10]. Poor bioactivity of CSH cement inhibits it from forming a chemical bond with bone tissue at the early stage of therapy [8, 11]. Moreover, the fast resorption of CSH cement may negatively affect the bone regeneration [12, 13].

Among the various biodegradable materials, magnesium (Mg) and its alloys have been utilized as metallic materials for medical implants because of their unique biocompatibility, nontoxicity, density, and elastic modulus similar to those of human bone and stimulatory effects on new bone formation [14–18]. However, in the physiological environment, they are corroded rapidly and thus lose the mechanical properties [19]. In order to lower the biodegradation ratio of Mg alloys, approaches like alkali-heat treatment [18], fluoride conversion coating [20], and plasma immersion ion implantation have been studied [21]. However, biocompatibility should be considered when graft material is used in human body. Considering the advantages and disadvantages of CSH and Mg alloys, it is proposed that the addition of Mg into CSH might result in a composite cement with improved properties and the Mg/CSH composite scaffold could be used to repair bone defects.

In the present study, a composite cement was prepared by adding Mg into CSH; meanwhile the setting time, injectability, mechanical properties,* in vitro* bioactivity, and biocompatibility with canine bone marrow stromal cells (cBMSCs) were evaluated. Moreover,* in vivo* bone formation capacity was also investigated by implanting the Mg/CSH scaffold into a canine tibia defect model. It was expected that the novel Mg/CSH composite cement could be potentially used for the clinical repair of bone defects.

## 2. Materials and Methods

### 2.1. Sample Preparation and Characterization

Mg powders were primarily prepared by a fluoride coating and micro-arc oxidation treatment (treated by Institute of Metal Research, Chinese Academy of Sciences, Shenyang, China). The Mg/CSH powder was prepared by adding Mg powders (weight ratio: 0%, 10%, and 20%) into the CSH powder (purchased from Allgens Co., Ltd., China). Then, deionized water was added with a liquid to powder ratio of 0.6 mL/g to moisten these powders. The mixtures were stirred within 60 s to form homogeneous pastes, transferred into Teflon molds (Φ 10 mm × 20 mm) and then stored at 37°C for 24 h; finally the hardened 10% Mg/CSH composite, 20% Mg/CSH composite, and pure CSH cements were obtained. The phase compositions of these samples were characterized by using X-ray diffraction (XRD; D8 Advance, Bruker (Beijing) Technology Co., Ltd., China) based on monochromated CuK_*α*_ radiation (*λ* = 1.5405 Å, 120 mA, 40 kV) in a continuous scan mode. The 2*θ* range was from 10° to 90° at a scanning speed of 8°/min.

### 2.2. Setting Time, Injectability, and Compressive Strength

The setting time of CSH and Mg/CSH composite pastes was measured with a Vicat needle (LeiYun Experimental Apparatus Manufacturing Co., Ltd., Shanghai, China) according to ISO9597-1989E. The initial setting time was defined as the time necessary for the light needle (228.6 g, *Ø* 5.067 mm) to plunge into the paste no longer leaving a visible print on the surface of the paste, while the final setting time was defined as the time necessary for the heavy needle (306 g, *Ø* 1.140 mm) to no longer leave a visible print on the surface of the paste. Five replicates were conducted for each group and the average value was calculated.

The injectability of CSH and composite pastes was tested by extruding a certain amount of paste through a 10 mL syringe with an opening nozzle (diameter: 2.8 mm), according to a method described previously [22]. Those pastes were filled into syringe, and the syringe was gently pressed at a speed of 15 mm/min until either pastes were completely extruded, or a maximum force of 100 N was reached. Then the weight of the extruded pastes was measured and the injectability was calculated by using the following formula: Injectability (%) = (Weight of extruded paste/Weight of total paste initially contained in syringe) × 100%.

Furthermore, the compressive strength of the hardened CSH and Mg/CSH composite samples (10 mm diameter × 20 mm high) was measured at a loading rate of 0.5 mm/min with a universal testing machine (ZWICK Co. Ltd., Germany) in a way similar to that described in ASTM D695-91. The measurements were performed five times for each group.

### 2.3. *In Vitro* Immersion Test in Simulated Body Fluid (SBF)

According to the method described by Kokubo [23], SBF was prepared, in which the degradation and bioactivity of CSH and Mg/CSH composite cements were evaluated. SBF consisted of 142.0 mM Na^+^, 5.0 mM K^+^, 1.5 mM Mg^2+^, 2.5 mM Ca^2+^, 148.8 mM C1^−^, 4.2 mM HCO_3_^−^, and 1.0 mM HPO_4_^2−^ and was buffered at pH 7.25 with 45 mM hydrochloric acid (HCl) and 50 mM trishydroxymethyl aminoethane ((CH_2_OH)_3_(CNH_2_)). After setting for 24 h, the hardened CSH and Mg/CSH composite specimens were immersed in SBF with a surface-area-to-volume ratio of 0.1 cm^2^/cm^3^ at 37°C for 21 days. The temperature of SBF was maintained by using a shaking water bath, and SBF was completely refreshed every day. For each group, five samples were removed from SBF after incubation for 2, 4, 7, 10, 14, and 21 days, respectively. At each time point, the specimens were gently rinsed with deionized water, dried in a 37°C oven for 24 h, and then weighed. To measure* in vitro* degradation, weight loss percentage was calculated by using the following formula: Degradation ratio = (*W*_0_ − *W*_*t*_)/*W*_0_ × 100%, where *W*_*t*_ and *W*_0_ represent the dry weights of the degraded specimen and the initial specimen, respectively.

Furthermore, in order to evaluate the* in vitro* bioactivity of samples, scanning electron microscopy (SEM) equipped with an energy dispersive X-ray detector (EDX; Tescan Ltd., Shanghai, China) was utilized. Additionally, the pH values of SBF were measured during the test using an electrolyte-type pH meter.

### 2.4. Cell Culture and Osteogenic Induction

After intravenous anesthesia with 5% sodium pentobarbital (0.5 mL/kg), 5 mL bone marrow was harvested from the iliac crests of an adult beagle dog, transferred into a 10 mL preheparinized centrifuge tube, and centrifuged to remove fat and heparin. Mononuclear cBMSCs were separated by percoll (1.073 g/mL, Sigma, USA) gradient centrifugation [24] and cultured in a complete medium containing low glucose-Dulbecco's modified Eagle's medium (L-DMEM; Gibco, USA) with 10% fetal bovine serum (FBS; Hyclone, USA) and 100 U/mL streptomycin at a density of 5 × 10^4^/cm^2^. Cells were incubated at 37°C in a humidified atmosphere containing 5% CO_2_. The culture medium was refreshed after 48–72 h and then every 2-3 days. When 80–90% cell confluence was reached, the cells were detached with 0.25% trypsin containing 0.01% EDTA (Invitrogen, USA) and then subcultured.

For cell osteogenic differentiation, the fourth-generation cBMSCs were cultured in medium containing high glucose-DMEM (H-DMEM, Life Technologies, Inc., USA) with 10% FBS, 100 nM dexamethasone (Sigma-Aldrich Co. Ltd., USA), 10 mM *β*-sodium glycerophosphate (Sigma, USA), and 200 *μ*M ascorbic acid (Sigma-Aldrich Co. Ltd., USA). The medium was replaced every 2 days. All animal care and procedures were done in accordance with the Animal Care Guidelines from Chinese People's Liberation Army General Hospital's Animal ethics committee.

### 2.5. *In Vitro* Biocompatibility

#### 2.5.1. Cytotoxicity Assay

The cytotoxicity assay was conducted by culturing cBMSCs in the extracts of the CSH and Mg/CSH composite specimens and using a 3-(4,5-dimethylthiazol-2-yl)-2,5-diphenyltetrazolium bromide (MTT) (Sigma-Aldrich Co. Ltd., USA) quantitative proliferation assay. According to ISO 10993-1: Biological evaluation of medical devices Part 1: Evaluation and testing within a risk management process, 0.2 g/mL extracts were prepared by soaking cements in cell culture medium for 1 day at 37°C, 5% CO_2_ (10 mL extract for each cement). The third-generation cBMSCs after osteogenic differentiation were detached by 0.25% trypsin, and then cell suspension was transferred to 96-well plates (volume: 200 *μ*L/well, density: 5 × 10^4^/mL). Six wells were set for each cement group. After incubation at 37°C in a humidified atmosphere of 5% CO_2_ for 24 h, cBMSCs were cultured in the presence of 100 *μ*L extracts, and afterwards the medium was refreshed every 2 days. After 2, 4, 6, and 8 days, 20 *μ*L MTT (5 g/L) was added into each well, and cBMSCs were incubated for further 4 h. Subsequently, the supernatant in each well was carefully removed and 150 *μ*L dimethyl sulfoxide was added. After shaking for 10 min, optical density (OD) values at 490 nm were evaluated by using a microplate reader.

#### 2.5.2. Proliferation of cBMSCs and Cell Cycle Test Based on Flow Cytometry

The proliferation of cBMSCs seeded on the pure CSH and Mg/CSH composite cements was evaluated by using cell cycle test based on flow cytometry. After 24, 48, and 72 h of incubations, cBMSCs were collected with trypsinization and centrifugation at 1000 rpm and washed twice with PBS. Then, cBMSCs were resuspended in PBS, fixed in 70% ethanol at 4°C for overnight. Finally, samples were centrifuged and stained with 0.5 mL hypotonic solution containing 50 *μ*g/mL PI, 0.2% Triton X-100, and 100 *μ*g/mL RNase A for 30 min in dark at 4°C. The numbers of cell at different phases of cell cycle were analyzed by using a flow cytometer at 488 nm excitation band. The proliferation index of cell was calculated as follows: Proliferation index = (S + G2M)/(G0G1 + S + G2M) × 100%.

#### 2.5.3. Osteogenic Differentiation and Alkaline Phosphatase (ALP) Biosynthesis

For osteogenic differentiation, the biosynthesis of ALP in cBMSCs cultured on the pure CSH and Mg/CSH composite cements was measured. After 7, 14, 21, and 28 days of incubation, culture medium was wiped off, and cBMSCs were washed twice with PBS. Approximately 1 mL of cell lysis buffer containing 0.2% Triton X-100 was added to lyse cBMSCs at room temperature, and cell lysate was obtained. After centrifugation, exactly 100 *μ*L of supernatant was moved to 96-well plates, and 100 *μ*L p-nitrophenyl phosphate (pNPP, 1 mg/mL) substrate solution was added. After incubation for 30 min at 37°C, the reaction was quenched by adding 50 *μ*L NaOH, and the absorbance at 405 nm was quantified with a plate reader. Each test was conducted five times.

#### 2.5.4. Cellular Morphology and Attachment of cBMSCs

The cellular morphology and attachment of cBMSCs were examined based on direct visualization under SEM. Firstly, cells were attached to the specimens for 3, 7, and 14 days at 37°C in an atmosphere of 100% humidity and 5% CO_2_. Subsequently, the cell-cement constructs were washed twice with PBS and fixed with 2.5% glutaraldehyde solution for 2 h at 4°C. Then, the fixed constructs were rinsed three times with PBS and dehydrated in graded ethanol (v/v: 50, 60, 70, 80, 90, and 100%). After being CO_2_-dried overnight in a desiccator, samples were sputtered with gold prior to SEM observation.

#### 2.5.5. Expressions of Osteogenesis and Attachment-Related Genes

The relative expressions of osteogenesis and attachment-related genes in cBMSCs were examined by using real-time RT-PCR (Thermo, USA). After being cultured on the hardened constructs for 8, 24, and 48 h, cBMSCs were homogenized in Trizol Reagent. Total RNA was extracted and reversely transcribed into cDNA according to the manufacturer's instructions. The sequences of primers for type I collagen (Coll I), integrin *β*1, and *β*-actin are shown in Table 1. The SYBR Green real-time PCR assay was carried out to measure the expression of genes according to its manual. Relative expression of each target gene was evaluated via the 2^−ΔΔCT^ method [25].

### 2.6. Implantation* In Vivo*

Totally, 24 healthy adult male beagle dogs weighing 16 ± 1 kg were randomly divided into 4 groups (6 dogs for each type of implant). The dogs were anesthetized through 3% pentobarbital (general anaesthesia) and 1% lidocaine (operative region anaesthesia) and placed in a supine position. Under sterile conditions, a 3 cm longitudinal skin incision was performed at the anteromedial aspect of left tibia. Skin and subcutaneous tissues were gently dissected down to periosteum, exposing the bone. Then, one tibial bone tunnel (3 mm diameter and 15 mm length) was surgically produced. The defects were filled with 20% Mg/CSH, 10% Mg/CSH, or pure CSH construct, and bone defects of the control group were left unfilled. All wounds were routinely sutured and penicillin (25,000 U/kg) was injected into all animals for 3 days. After surgery, the canines were kept caged freely and given usual regimen of food and water.

Animals were sacrificed 4 and 12 weeks after operation and tibia specimens were harvested and fixed in 4% paraformaldehyde. The local bone mineral densities (BMDs) were measured on a dual energy X-ray absorptiometry (DXA) system, and the new bone area fraction (BAF) was quantified 1 day and 4 and 8 weeks after implantation by using the following formula: BAF = AB/AT, where AB is the newly formed bone area and AT is the total material area. For histological analysis after 4, 8, and 12 weeks after surgery, the decalcified and undecalcified bone specimens were, respectively, embedded into paraffin and hard plastic. Tissue sections were stained with hematoxylin and eosin (H&E) and Masson's Trichrome stain, respectively, and then observed under a light microscope (Olympus BX51, Japan). Additionally, the tissue specimens (5 mm × 5 mm × 5 mm) around the implantation materials were removed and immediately immersed into RNAlater solution. Then, the total tissular RNA was extracted and reverse-transcribed into cDNA, and the transcription levels of osteogenesis-related canine osteopontin, bone morphogenetic protein-2 (BMP-2), and Coll I were measured by RT-PCR.

### 2.7. Statistical Analysis

Experimental data were expressed as means ± SD. The one-way and two-way ANOVA with Tukey's post hoc tests were applied to comparison analysis. Differences were considered statistically significant at *p* < 0.05.

## 3. Results

### 3.1. Characterization of Mg/CSH

**Figure 1:**
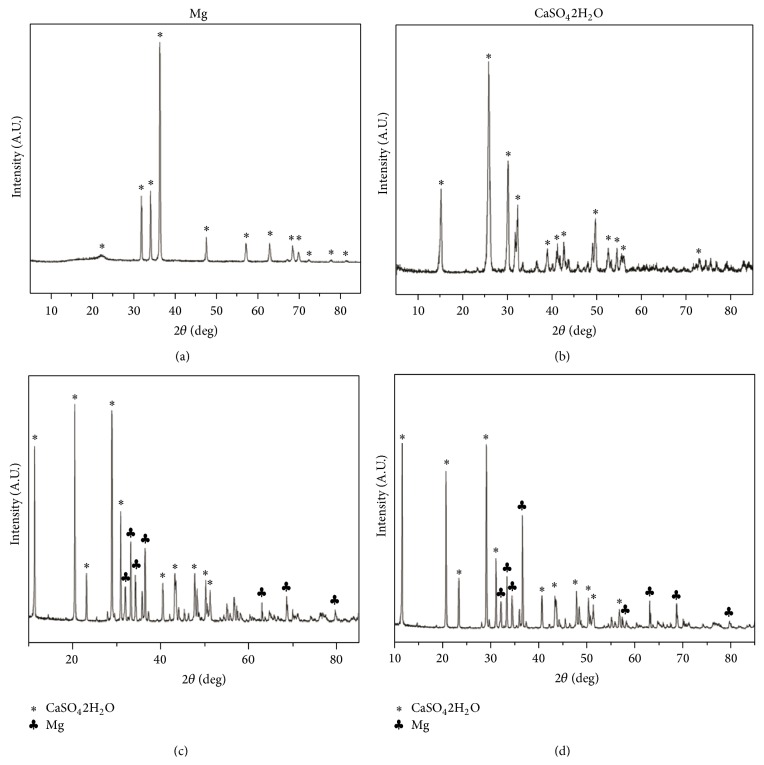
X-ray diffraction patterns after setting for 24 h. (a) Mg; (b) CSH; (c) 10% Mg/CSH composite; (d) 20% Mg/CSH composite. Mg: magnesium; CSH: calcium sulfate hemihydrate.

After setting for 24 h, the phase composition of the hardened Mg/CSH composite was characterized by using XRD. The CSH construct contained diffraction peaks of CaSO_4_·2H_2_O (Figure 1(b)), and a mixture of CaSO_4_·2H_2_O and Mg could be seen in the XRD patterns of the Mg/CSH composites with 10% and 20% Mg (Figures 1(c) and 1(d)). The presence of CaSO_4_·2H_2_O could be attributed to the reaction of CaSO_4_·1/2H_2_O and H_2_O.

### 3.2. Setting Time, Injectability, and Compressive Strength of Mg/CSH

**Figure 2:**
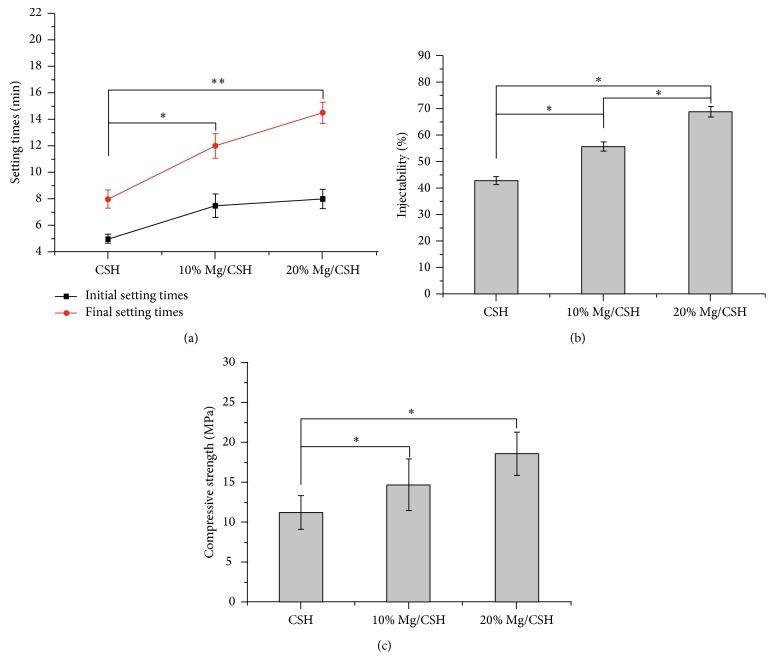
The setting time, injectability, and compressive strength of CSH and Mg/CSH composite specimens. (a) The initial and final setting time; (b) injectability; (c) compressive strength after setting for 24 h. ^*∗*^ *p* < 0.05 and ^*∗∗*^ *p* < 0.01 indicate that the setting time, injectability, and compressive strength of Mg/CSH composite specimens were significantly different from those of CSH. The bars on the graphs are standard deviations. Mg: magnesium; CSH: calcium sulfate hemihydrate.

The initial and final setting time of 10% and 20% Mg/CSH composites were significantly higher than those of pure CSH, and setting time increased with increasing weight ratio of Mg. The longest setting time (*p* < 0.01) was observed in 20% Mg/CSH composite with the initial and final setting time of 8 ± 0.72 min and 14.5 ± 0.8 min, respectively (Figure 2(a)). The injectability of Mg/CSH composite pastes was significantly improved in comparison with that of CSH paste (*p* < 0.05). Moreover, the injectability of Mg/CSH composite pastes dramatically increased with the increase of Mg content, and 20% Mg/CSH composite paste exhibited the highest injectability (69 ± 2%, *p* < 0.05) (Figure 2(b)). After setting for 24 h, the compressive strength of the hardened constructs also significantly rose (*p* < 0.05) with the increase of Mg content and reached a maximum value of 18.6 ± 2.7 MPa in 20% Mg/CSH composite, while there is no significant difference (*p* > 0.05) between 10% Mg/CSH and 20% Mg/CSH composites (Figure 2(c)).

### 3.3. *In Vitro* Bioactivity, Degradation, and pH Value Change in SBF

**Figure 3:**
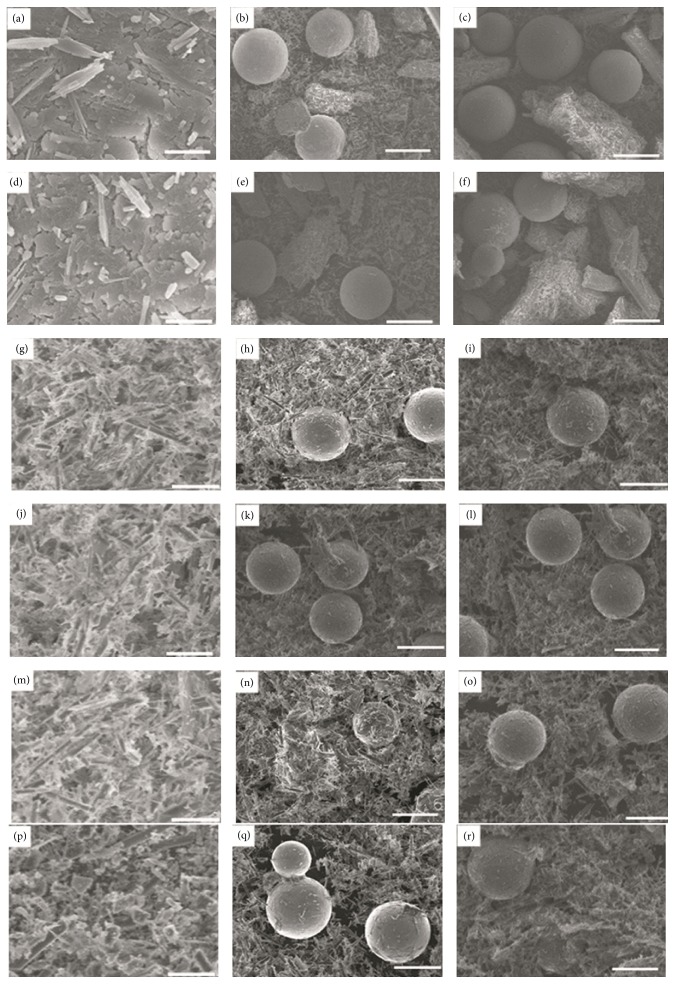
Scanning electron microscope micrographs of the CSH and Mg/CSH composite specimens after soaking in simulated body fluid for different time. CSH (a), 10% Mg/CSH (b) and 20% Mg/CSH (c) for after 2 days. CSH (d), 10% Mg/CSH (e) and 20% Mg/CSH (f) for after 4 days. CSH (g), 10% Mg/CSH (h) and 20% Mg/CSH (i) for after 7 days. CSH (j), 10% Mg/CSH (k) and 20% Mg/CSH (l) for after 10 days. CSH (m), 10% Mg/CSH (n) and 20% Mg/CSH (o) for after 14 days. CSH (p), 10% Mg/CSH (q) and 20% Mg/CSH (r) for after 21 days. Magnification: ×500. Scale bar: 50 *μ*m.

**Figure 4:**
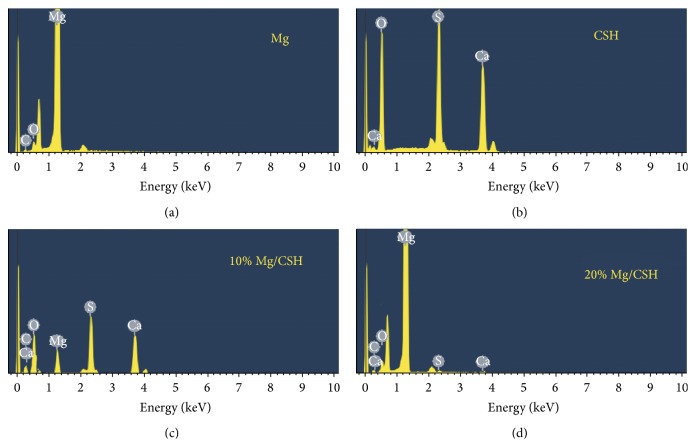
EDX analysis of specimens immersed in simulated body fluid for 21 days. (a) Mg; (b) CSH; (c) 10% Mg/CSH composite; (d) 20% Mg/CSH composite. EDX: energy dispersive X-ray detector; Mg: magnesium; CSH: calcium sulfate hemihydrate.

SEM micrographs of surface showed the influence of SBF on the microstructure of materials (Figure 3). After soaking for 2 and 4 days, vast ribbed crystals were observed in the CSH specimens (Figures 3(a) and 3(d)). With long immersion time, a sediment layer was formed on the surface (Figures 3(g), 3(j), 3(m), and 3(p)). For the Mg/CSH composite specimens, ball-like Mg particles and CaSO_4_ crystals were displayed after 2 and 4 days of immersion (Figures 3(b)-3(c) and 3(e)-3(f)). Subsequently, many sediments formed and further congregated to form a layer on the surface of sample, while Mg particles showed no obvious change (Figures 3(e)-3(f), 3(h)-3(i), 3(k)-3(l), 3(n)-3(o), and 3(q)-3(r)). There was no obvious difference between the surfaces of 10% and 20% Mg/CSH composites after immersion. EDX indicated that the surfaces of pure Mg contained Mg, C, and O ions (Figure 4(a)), and CSH cement had some O, S, and Ca (Figure 4(b)). The 10% and 20% Mg/CSH composite samples consisted mainly of Mg, C, O, S, and Ca (Figures 4(c) and 4(d)) after soaking for 21 days.

**Figure 5:**
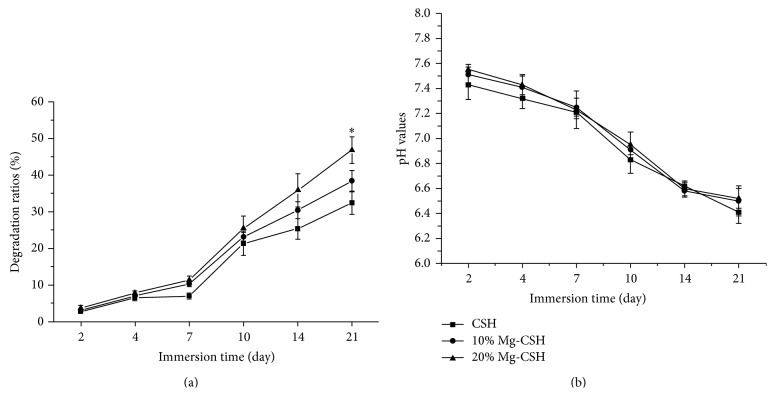
Degradation ratios of CSH and Mg/CSH composite specimens immersed for 2, 4, 7, 10, 14, and 21 days. (a) Simulated body fluid; (b) pH values of the environment. *∗* indicates that degradation ratios of the Mg/CSH composite specimens were significantly different from those of CSH (*p* < 0.05). The bars on the graphs are standard deviations. Mg: magnesium; CSH: calcium sulfate hemihydrate.

The degradation ratios of the samples were characterized by the weight loss ratios after soaking in SBF for various time periods. It can be seen that there were no significant differences (*p* > 0.05) in the degradation ratios among all specimens from 2 to 14 days, while the degradation ratio of 20% Mg/CSH composite was significantly higher (*p* < 0.05) than that of other constructs after 21 days of soaking (Figure 5(a)). During immersion in SBF, the samples led to an acid environment, causing a decrease in the pH values of SBF. However, no remarkable difference (*p* > 0.05) was observed among the three kinds of construct at selected time points (Figure 5(b)).

### 3.4. Viability, Proliferation, Differentiation, Attachment, and Morphology of cBMSCs after Incubation with Biomaterials

**Figure 6:**
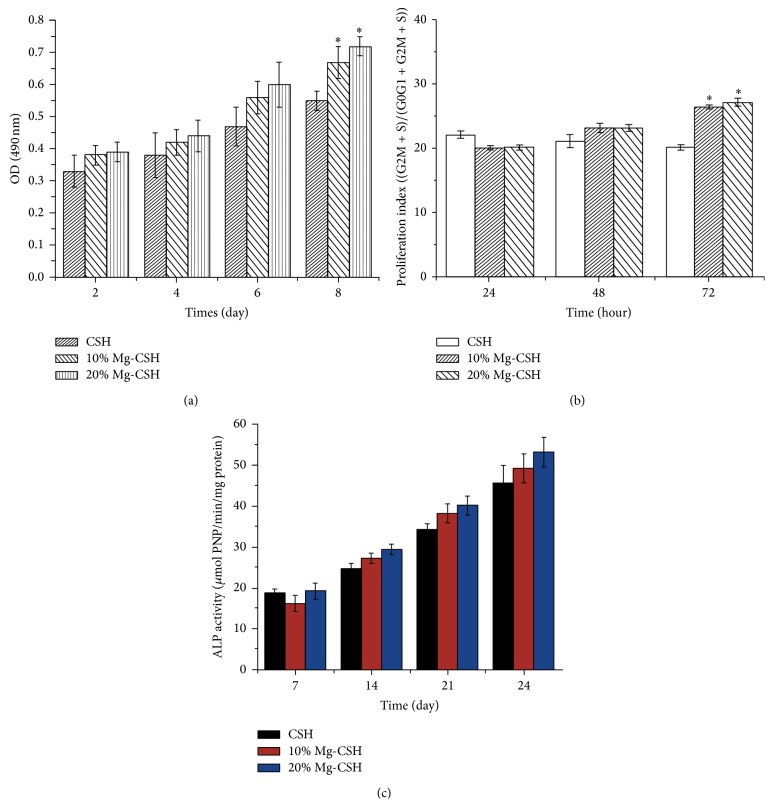
Cell viability, proliferation index, and alkaline phosphatase activity. (a) Cell viability incubated with the extraction fluids of CSH and Mg/CSH composite specimens after 2, 4, 6, and 8 days; (b) proliferation index cultured on CSH and Mg/CSH composite specimens after 24, 48, and 72 h; (c) ALP activity of cells cultured on CSH and Mg/CSH composite specimens after 7, 14, 21, and 24 days. *∗* indicates that the cell viability and proliferation index of Mg/CSH composite specimens were significantly different from those of CSH (*p* < 0.05). The bars on the graphs are standard deviations. Mg: magnesium; CSH: calcium sulfate hemihydrate; ALP: alkaline phosphatase.

Changes of the viability of cBMSCs cultured in different extracts were assessed through MTT assay. It was observed that the OD values in all extracts increased with time, indicating that three constructs caused no significant cytotoxicity against cells during various time periods. However, the viability of cBMSCs in the extracts of both 10% and 20% Mg/CSH composite specimens was significantly higher (*p* < 0.05) than that of cBMSCs in the extracts of pure CSH after incubating for 8 days (Figure 6(a)).

No significant difference (*p* > 0.05) was observed among the proliferation indexes of cBMSCs cultured on different materials for 24 and 48 h. However, the Mg/CSH composites (10% and 20%) could significantly (*p* < 0.05) increase cell proliferation when compared with the CSH after 72 h of culture (Figure 6(b)).

Cellular differentiation was evaluated by testing the ALP activity of cBMSCs cultured on construct specimens for 7, 14, 21, and 24 days. The ALP activities of cells grown on all three materials were elevated with time, while there was no significant difference (*p* > 0.05) among ALP activities of cBMSCs in three groups at a certain time point (Figure 6(c)).

**Figure 7:**
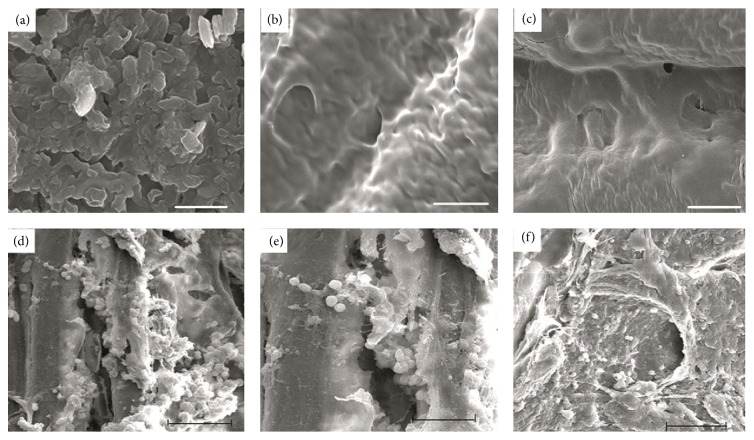
Scanning electron microscope micrographs showing the morphological features of cells cultured on CSH and Mg/CSH composite specimens. Images of cells cultured on CSH (a) and 10% Mg/CSH composite specimens (d) for 3 days; images of cells cultured on CSH (b) and 10% Mg/CSH composite specimens (e) for 7 days; images of cells cultured on CSH (c) and 10% Mg/CSH composite specimens (f) for 14 days. Magnification: ×500. Scale bar: 50 *μ*m. Mg: magnesium; CSH: calcium sulfate hemihydrate.

The cells firmly attached and exhibited morphologically normal appearance on the surface of 10% Mg/CSH and CSH constructs after 3 days of culture (Figures 7(a) and 7(d)). Cells extended and spread well after 7 days of culture (Figures 7(b) and 7(e)), ultimately forming a confluent layer with intimate attachment to the material surface in 14 days (Figures 7(c) and 7(f)).

**Figure 8:**
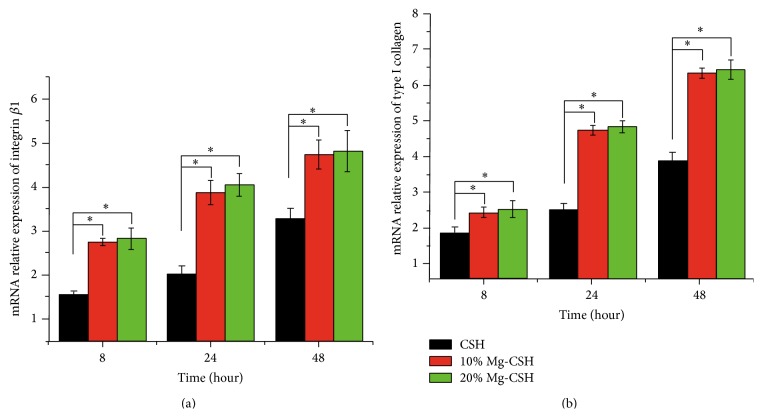
mRNA relative expressions of integrin *β*1 and type I collagen. (a) Integrin *β*1; (b) type I collagen. Expression levels were normalized according to housekeeping gene (*β*-actin). Significant differences between Mg/CSH composite specimens and pure CSH were found (^*∗*^ *p* < 0.05). The bars on the graphs are standard deviations. Mg: magnesium; CSH: calcium sulfate hemihydrate.

The relative expression values of integrin *β*1 gene were dramatically increased (*p* < 0.05) in cBMSCs cultured on Mg/CSH composites (10% and 20%) in comparison with CSH construct, while no significant difference (*p* > 0.05) was observed between 10% and 20% Mg/CSH at 8, 24, and 48 h (Figure 8(a)). The Coll I expressions showed similar trends (Figure 8(b)).

### 3.5. *In Vivo* DXA Analysis

**Figure 9:**
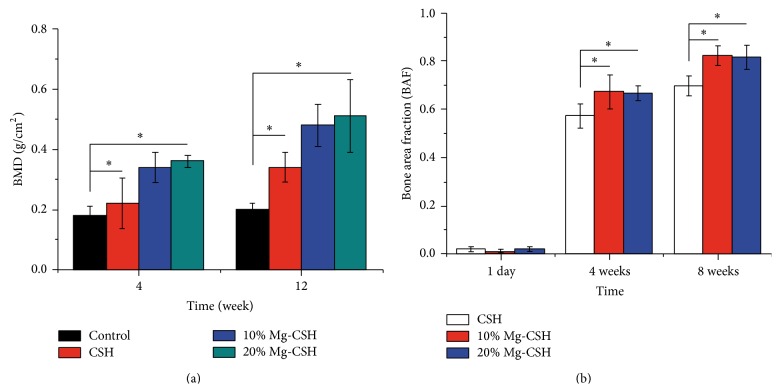
Quantitative analysis of BMD and BAF based on a dual energy X-ray absorptiometry system. (a) The local BMD after implantation for 4 and 12 weeks; (b) the BAF for 1 day, 4 weeks, and 12 weeks. *∗* represents significant differences between Mg/CSH composite specimens and pure CSH (*p* < 0.05). The bars on the graphs are standard deviations. BMD: bone mineral density; BAF: new bone area fraction; Mg: magnesium; CSH: calcium sulfate hemihydrate.

To quantify the calcification of repaired tibia, BMDs of all animals were measured on DXA 4 and 12 weeks after operation. The Mg/CSH grafts showed higher (*p* < 0.05) BMD values than CSH and control groups at both 4 and 12 weeks. However, there was no marked variation (*p* > 0.05) between 10% and 20% Mg/CSH grafts (Figure 9(a)). Meanwhile, BAF was applied to evaluate the newly formed bone after surgery for 1 d and 4 and 8 weeks. The BAFs of Mg/CSH composite graft were significantly higher (*p* < 0.05) than that of CSH at both 4 and 8 weeks (Figure 9(b)).

### 3.6. *In Vivo* Histological Analysis

**Figure 10:**
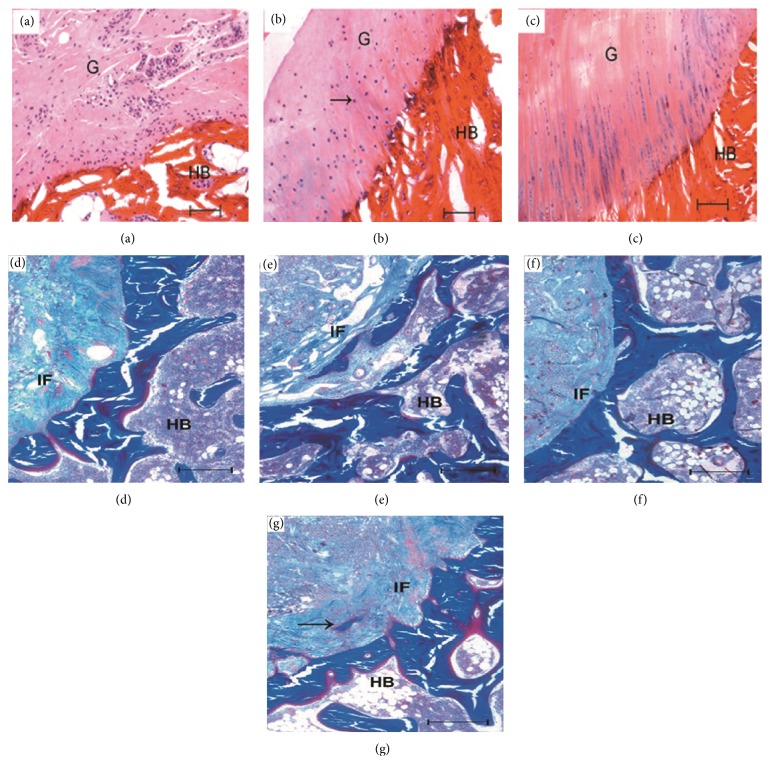
Histology photomicrographs after hematoxylin and eosin staining and Masson's Trichrome staining of the decalcified bone defect sections after implantation. (a) 10% Mg/CSH for 4 weeks, hematoxylin and eosin staining; (b) 10% Mg/CSH for 8 weeks, hematoxylin and eosin staining; (c) 10% Mg/CSH for 16 weeks, hematoxylin and eosin staining; (d) CSH for 4 weeks, Masson's Trichrome staining; (f) CSH for 8 weeks, Masson's Trichrome staining; (e) 10% Mg/CSH for 4 weeks, Masson's Trichrome staining; (g) 10% Mg/CSH for 8 weeks, Masson's Trichrome staining. Magnification: ×200; scale bar for (a)–(c): 200 *μ*m; scale bar for (d)–(g): 500 *μ*m. Abbreviations and signs used: graft (G), host bone (HB), interface of new bone and host bone (IF), magnesium (Mg), and calcium sulfate hemihydrate (CSH). Black arrows indicated the new trabeculae.

After 4 weeks' implantation, new chondrocytes appeared in many areas of the 10% Mg/CSH composite implant and some inflammatory cells could be seen in the center of bone defect area (Figure 10(a)). The cellular differentiation appeared at 8 weeks (Figure 10(b)) and more cells gathered in fascicles at the interface between the implant materials and the host bone after 8 weeks (Figure 10(c)). Masson staining showed no new bone tissue was observed at the interface of CSH implant after 4 (Figure 10(d)) and 8 (Figure 10(e)) weeks, while new trabeculae could be seen in the implanted 10% Mg/CSH composite section at 8 weeks (Figure 10(g)).

**Figure 11:**
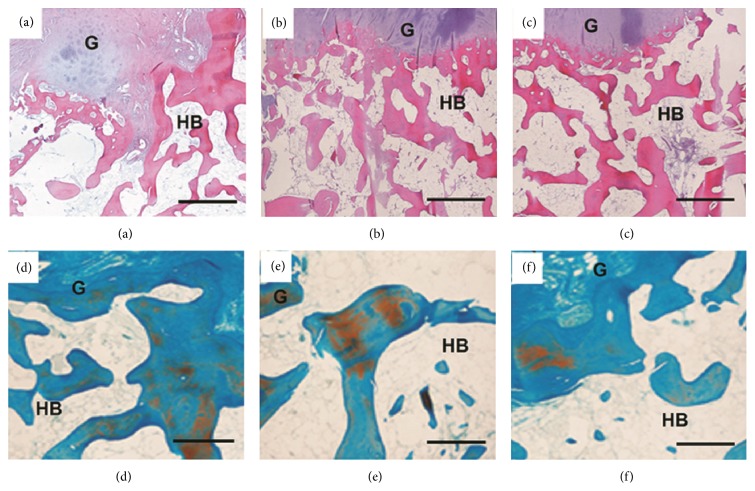
Histology photomicrographs of hematoxylin and eosin staining and Masson's Trichrome staining of the undecalcified bone defect sections after implantation. (a) Hematoxylin and eosin staining, 10% Mg/CSH for 4 weeks; (b) hematoxylin and eosin staining, 10% Mg/CSH for 8 weeks; (c) hematoxylin and eosin staining, 10% Mg/CSH for 16 weeks; (d) Masson's Trichrome staining, 10% Mg/CSH for 4 weeks; (e) Masson's Trichrome staining, 10% Mg/CSH for 8 weeks; (f) Masson's Trichrome staining, 10% Mg/CSH for 16 weeks. Scale bar: 200 *μ*m. Abbreviations and signs used: graft (G), host bone (HB), magnesium (Mg), and calcium sulfate hemihydrate (CSH).

Undecalcified bone histology can preferably demonstrate cellular components of bone, bone turnover, and formation. New chondrocytes were still seen in the implant areas of 10% Mg/CSH at 4–16 weeks (Figures 11(a)–11(c)) in accordance with the decalcified staining results. Moreover, the boundaries between normal surrounding tissue and the composite specimens were gradually indistinct due to biodegradation, and the residual materials were surrounded by areas of newly formed bone tissue during 4–12 weeks (Figures 11(d)–11(f)).

### 3.7. Analysis of Gene Expression

**Figure 12:**
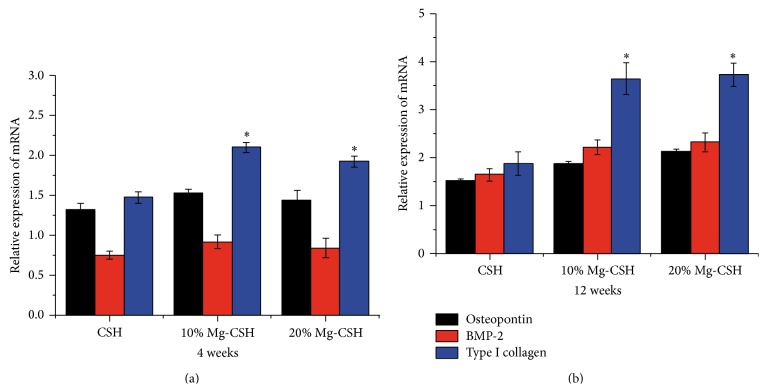
mRNA relative expressions of osteopontin, BMP-2, and type I collagen after implantation. (a) After implantation for 4; (b) after implantation for 12 weeks. Expression levels are normalized according to housekeeping gene (*β*-actin). *∗* stands for significant differences of type I collagen relative expressions between Mg/CSH composite specimens and pure CSH (*p* < 0.05). The bars on the graphs are standard deviations. Mg: magnesium; CSH: calcium sulfate hemihydrate; BMP-2: bone morphogenetic protein-2.

The relative expression levels of osteopontin among the tissues around three graft materials exhibited no significant difference (*p* > 0.05) after implantation for 4 and 12 weeks, which was similar to BMP-2 expression. Nevertheless, the expression levels of Coll I in both Mg/CSH composite groups were significantly higher (*p* < 0.05) than that of CSH construct at 4 and 12 weeks (Figures 12(a) and 12(b)).

## 4. Discussion

Because bone is needed for mineral reserve, locomotion, load bearing, and protection of internal organs, bone defect causes disability and represents a medical and socioeconomic challenge. Tissue engineering is playing a critical role in bone regeneration [3]. An ideal bone grafting material should not only possess mechanical stability and excellent bioactivity, but also have osteoconductivity and osteoinductivity [26–28]. In the present study, Mg/CSH composite cement showed a prolonged setting time with improved injectability and enhanced mechanical strength due to the addition of Mg in comparison with CSH alone. In addition, a significantly improved degradability and promoting effect on the proliferation and osteogenic differentiation of cBMSCs* in vitro* were also exhibited by Mg/CSH composite constructs. Histological evaluation and analyses of DXA and gene expression indicated that Mg/CSH could enhance the efficiency of new bone formation in comparison with CSH. This implied that this novel injectable bone scaffold (Mg/CSH composite) would have a great potential for bone repair in tissue engineering.

The applicability of a bone cement biomaterial is largely dependent on its self-setting characteristics including injectability and setting time [29]. Additionally, in clinical applications, the cement must be extruded and applied before its initial setting start during operation [30]. In comparison with pure CSH paste, which had short initial and final setting time, Mg/CSH composite pastes showed a relatively prolonged setting time. The prolonged setting time improved the injectability of cement and could result in an obvious advantage for surgeons by allowing more time to work before paste starts setting. Mechanical property of the hardened cement is another important index for the clinical applications of bone materials [29]. Nevertheless, previous studies have suggested that pure CSH cement fails to meet this index because of its poor and nearly constant mechanical strength [9, 31]. In contrast, the compressive strength of Mg/CSH composite constructs increased along with the increase of Mg content (in comparison with that of pure CSH), and this would provide a much better mechanical support for the defect site during the bone regeneration process.

Bioactivity is defined as the ability of biomaterials to develop an adherent, direct, and strong bonding with the bone tissue [32]. Reportedly, CSH cement always lacks the capability of forming a chemical bond with bone tissue at the early stage of the implantation due to its poor bioactivity [8, 11–13]. However, in the present study, SEM and EDX analyses suggested that apatite deposition could not be observed on the surface of all the pure CSH and Mg/CSH composite within 21 days after soaking in SBF. It was indicated that the addition of Mg might not contribute to the tendency of the CSH to form bone-like apatite in SBF. However, further studies are needed to improve the bioactivity of Mg/CSH composite constructs. Moreover, the biomaterial should be degradable and gradually replaced by newly formed bone tissue [33]. The proper degradability of a biomaterial in a physiological environment is one of the most important characteristics [28]. In our present study, Mg/CSH composite cements exhibited a significantly higher degradability than pure CSH, and 20% Mg/CSH had a better degradability property after 21 days of immersion, suggesting that the degradation ratio of composite cement could be adjusted by the addition of Mg.

It is generally accepted that* in vitro* cellular responses to biomaterials, including cell attachment, proliferation, and differentiation, are also main components of new bone repair ability of biomaterials [34]. The osteogenic potential of BMSCs has been demonstrated both* in vitro* and* in vivo* [35–37]. Therefore, BMSCs have frequently been utilized to evaluate the biocompatibility of synthetic materials for bone engineering. In the present study, Mg/CSH composite pastes did not induce significant cytotoxicity, and they facilitated cBMSCs proliferation in comparison with the pure CSH cement. Among all bone fiber collagen molecules, Coll I is the most important kind of collagen fiber and considered as a necessary gene for bone formation and remodeling, which also can provide fiber reinforcement to the cement [28]. Furthermore, integrin plays an important role in the process of cell adhesion and extension and is an important protein connecting osteoblasts and bone substitutes, which is also a necessary specific gene in cell adhesion and osteogenesis [38]. In the present study, the enhanced expression of Coll I and integrin *β*1 in cBMSCs cultured on Mg/CSH composite constructs demonstrated the distinguished ability of cell adhesion and osteogenesis by adding Mg, in comparison with pure CSH. Therefore, the* in vitro* results indicated that this composite exhibited favorable biocompatibility by improving cell attachment and stimulating cell proliferation and differentiation.

Unlike autografts which have limited supply and significant potential risk of nerve damage, infecting, disease transmission, and immune response [28, 39], bone cement substitutes are promising approaches for bone regeneration [3]. Reportedly, CSH and Mg have been widely used in the clinic as a bone regeneration scaffold, while a variety of disadvantages remain. In the* in vivo* study, BMDs of Mg/CSH graft were significantly higher than that of pure CSH at 4 and 12 weeks after implantation. Moreover, the increased BAF indicated that more new bone was formed in Mg/CSH composite groups in comparison with CSH 4 and 8 weeks after graft. Histological evaluation also revealed that the new chondrocytes, trabeculae, and mature ossein appeared at the defect area, the boundaries between normal surrounding tissue and the composite were gradually indistinct due to biodegradation, and the residual materials were surrounded by areas of newly formed bone tissue during 8 weeks after implantation of composite cement. On the contrary, no new bone tissue was observed at the interface of CSH implant after 8 weeks, and the boundaries between normal surrounding tissue and CSH were distinct. It may be attributed to the addition of Mg which can accelerate the growth of new bone tissue as previously reported [40, 41]. Therefore, these* in vivo* results demonstrated that Mg/CSH composites exhibited not only faster biodegradability but also more effective osteogenesis and osteointegration at bone defect area than pure CSH cement.

## 5. Conclusions

A novel injectable Mg/CSH composite was developed by incorporating Mg coated with fluoride into CSH in this study. Mg/CSH composites showed a prolonged setting time with improved injectability. The mechanical strength and biodegradability of the Mg/CSH composite were improved. The Mg/CSH composite could promote the attachment, proliferation, and differentiation of canine cBMSCs and exhibit excellent biocompatibility without cytotoxicity. Additionally, the Mg/CSH composite implant also showed effective osteogenesis and osteointegration. In conclusion, this new kind of injectable biomaterial with improved properties would develop a more promising tissue graft substitute for bone regeneration.

## Figures and Tables

**Table 1:** The sequences of the primers used for qRT-PCR.

Gene	Primer sequence
Integrin *β*1	F5′-GTGCTGAAGACTACCCCATC
	R5′-CTCCACAAAAGAGCCAAATC
Type I collagen	F5′-ATGGATGAGGAAACTGGC
	R5′-TCAAGGAAGGGCAAACG
Osteopontin	F5′-AACCACAGTTTTCACTGAAGTCGT
	R5′-TCCAAGTCCTCGCTGTCCAC
BMP-2	F5′-GGGTATCACGCCTTTTACTGC
	R5′-TCGGAATCTTAGAGTTCACGGA
*β*-actin	F5′-GTGATGGTGGGCATGGGTC
	R5′-GATTCGTGCTCGATGGGGTA
